# Two-dimensional self-assembly and co-assembly of two tetracarboxylic acid derivatives investigated by STM†

**DOI:** 10.1039/d3na00389b

**Published:** 2023-08-03

**Authors:** Xuan Peng, Linlin Gan, Wenchao Zhai, Xiaoling Chen, Ke Deng, Wubiao Duan, Wei Li, Qingdao Zeng

**Affiliations:** a CAS Key Laboratory of Standardization and Measurement for Nanotechnology, CAS Center for Excellence in Nanoscience, National Center for Nanoscience and Technology (NCNST) Beijing 100190 China kdeng@nanoctr.cn zengqd@nanoctr.cn; b Center of Materials Science and Optoelectronics Engineering, University of Chinese Academy of Sciences Beijing 100049 China; c Department of Chemistry, School of Science, Beijing Jiaotong University Beijing 100044 China wbduan@bjtu.edu.cn; d School of Science, Nanchang Institute of Technology Nanchang 330099 China liweidting@nit.edu.cn

## Abstract

In this work, the two-dimensional self-assembly and co-assembly behaviors of two tetracarboxylic acid derivatives (H_4_BDETP and H_4_BTB) were investigated by scanning tunneling microscopy (STM). H_4_BDETP molecules self-assembled into linear nanostructures, and H_4_BTB molecules formed lamellar and tetragonal nanostructures. The formation of a H_4_BDETP/H_4_BTB co-assembly nanostructure was closely related to the deposition sequence of H_4_BDETP and H_4_BTB on highly oriented pyrolytic graphite (HOPG). The introduction of H_4_BTB into the self-assembly system of H_4_BDETP resulted in the emergence of the H_4_BDETP/H_4_BTB nanostructure, while the addition of H_4_BDETP had no effect on the self-assembly system of H_4_BTB and a H_4_BDETP/H_4_BTB co-assembly nanostructure was not obtained.

## Abstract 2

In this article, the hydrogen-bonded self-assembly nanostructures of two tetracarboxylic acid derivatives and the effect of the deposition sequence on their co-assembly nanostructure were investigated by STM.

## Graphical abstract

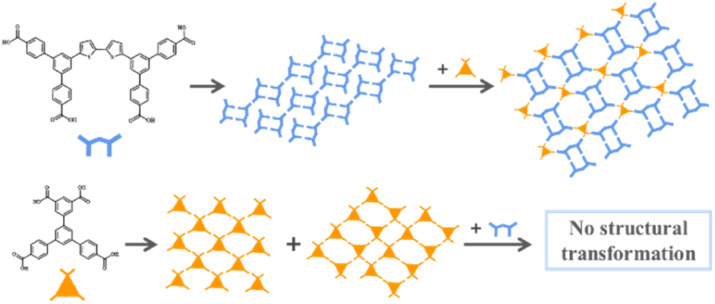

## 1. Introduction

Supramolecular self-assembly has been a wide concern in many fields such as nanopattern fabrication and heterogeneous nanomaterials ascribed to its ability to guide simple molecular components to spontaneously form complicated and well-organized molecular aggregation states *via* molecular non-covalent interactions.^1–4^ Supramolecular self-assembly is promising in the preparation of diverse functional nanostructures through designing the chemical structures of functional building blocks at the molecular level and controlling the connection of building blocks. The precise construction of multi-component self-assembly systems anticipated to possess functions that cannot be achieved using mono-component systems has attracted attention and yet remains challenging.^5,6^ The fabrication of multi-component nanostructures is affected not only by the complementarity of the chemically distinct molecular building blocks in spatial size and shape, but also by the uncontrollable connection between building blocks attributed to their abundant binding sites.^7,8^ The research on two-dimensional assembly systems at the molecular level is conducive to explore the characteristics and mechanism of molecular assembly and provides guidance for the controllable preparation of complex hybrid nanostructures. Scanning tunneling microscopy (STM) can provide information about self-assembly morphology at the molecular level, which is helpful for investigating the intermolecular interaction mode and the laws of molecular self-assembly.^9–11^

**Scheme 1:**
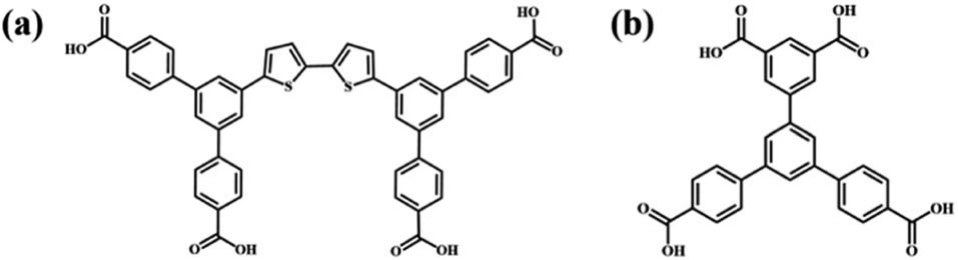
Chemical structures of (a) H_4_BDETP and (b) H_4_BTB.

As one of the non-covalent interactions, a hydrogen bond is supposed to be a powerful driving force for inducing molecular self-assembly and the formed self-assembly nanostructures are more predictable attributed to its directivity and saturation. Functional groups including carboxyl groups, amino groups, cyan groups *etc.* are modified in the molecular structure to provide hydrogen bonding sites for intermolecular recognition.^12–17^ Among them, the modification of carboxyl groups at the ends of benzene rings is a commonly used method for constructing hydrogen-bonded supramolecular assembly systems.^18–20^ Molecular interaction sites are closely related to the number and substitution position of carboxyl groups, which influences the molecular symmetry and the intermolecular interaction mode and further the final self-assembly nanostructure.^21^ Earlier research studies mostly focused on the construction of binary nanostructures composed of carboxylic acid derivatives and pyridine derivatives, and it was found that the introduced pyridine derivatives could disrupt the O–H⋯O hydrogen bonds between carboxylic acid derivatives and formed stronger O–H⋯N hydrogen bonds with carboxylic acid derivatives.^22–26^ However, in addition to pyridine derivatives, external aromatic acid derivatives can also regulate the self-assembly structure of carboxylic acid derivatives. Initially, the interface co-assembly behavior of two C_3_-symmetric triacid derivatives, 1,3,5-tris(4-carboxyphenyl)benzene (BTB) and trimesic acid (TMA), in two solvents has been revealed by STM.^27^ Three binary hydrogen-bonded networks were obtained by adjusting the mixed solution concentration of BTB and TMA. However, BTB and TMA separately self-assembled into mono-component nanostructures and phase separation was observed when mixing them in a ultra-high vacuum on Au(111).^28^ Later, the regulatory effects of BTB and TMA on the self-assembly of other carboxylic acid derivatives were investigated. For instance, TMA can act as a bridging molecule to connect with low-symmetry carboxylic acid derivative H_4_OBDB (or H_4_ADDI molecules) *via* hydrogen bonds, thus forming two-component nanostructures with alternating arrangement between H_4_OBDB (or H_4_ADDI) and TMA molecules.^29^ Terephthalic acid (TPA) and TMA co-assembled into a rectangular nanostructure or compact nanostructure, depending on the substrate bias.^30^ Tetracarboxylic acid (PBPTTBA) and BTB formed a novel co-crystallized network driven by homomeric and heteromeric R^2^2(8) hydrogen bonds.^31^ In this work, C_2_-symmetric 5′-(4-carboxyphenyl)-[1,1′:3′,1′′-terphenyl]-3,4′′,5-tricarboxylic acid (H_4_BTB) which possesses a terminal isophthalic acid group different from BTB was introduced to regulate the self-assembly nanostructure of 5′,5′′′′-([2,2′-bithiophene]-5,5′-diyl)bis(([1,1′:3′,1′′-terphenyl]-4,4′′-dicarboxylic acid)) (H_4_BDETP). And their chemical structures are displayed in Scheme 1.

In this article, the mono-component and bi-component assembly systems of the two C_2_-symmetric tetracarboxylic acid derivatives (H_4_BDETP and H_4_BTB) at the 1-heptanoic acid/HOPG interface were studied by STM. H_4_BDETP molecules constituted dimeric building blocks and further aggregated into an ordered linear nanostructure based on hydrogen bonds. The addition of H_4_BTB destroyed the H_4_BDETP's linear structure and gave rise to the formation of a H_4_BDETP/H_4_BTB co-assembly nanostructure. H_4_BDETP's dimer building blocks were unaffected and H_4_BTB functioned as a bridging molecule to connect with H_4_BDETP's dimers through hydrogen bonds. Interestingly, the deposition sequence is crucial for the formation of the H_4_BDETP/H_4_BTB co-assembly nanostructure.

## 2. Experimental section

### 2.1 STM detection

H_4_BDETP and H_4_BTB were bought from Jilin Chinese Academy of Sciences – Yanshen Technology Co., Ltd and 1-heptanoic acid from J&K company. All samples were directly used without further purification here. H_4_BDETP and H_4_BTB were dissolved in 1-heptanoic acid solvent with a solution concentration around 10^−4^ mol L^−1^. Their solutions were respectively dropped on the freshly cleaved HOPG (grade ZYB, NTMDT, Russia) surface treated with Scotch tape and then probed using a Nanoscope IIIa scanning probe microscope (Bruker, USA). And a Pt/Ir (80/20) wire after mechanically cutting was employed as the STM tip. After observing the linear assembly nanostructure of H_4_BDETP, a droplet of H_4_BTB solution was dropped on HOPG to obtain the bi-component nanostructure and then detected by STM. To investigate the effect of the molecular deposition sequence on the co-assembly result, H_4_BDETP was deposited on the HOPG substrate covered by the self-assembly nanostructures of H_4_BTB followed by STM characterization. Detailed imaging conditions are provided in the titles of all figures.

### 2.2 Theoretical calculations

Theoretical analysis in this work was fulfilled using density functional theory (DFT) calculations offered by the DMol3 code.^32^ Periodic boundary conditions (PBCs) were adopted to present the 2D periodic alignment on graphite. In order to give a depiction of exchange and correlation, the Perdew and Wang parametrization of the local exchange correlation energy was employed in the local spin density approximation (LSDA).^33,34^ All-electron spin-unrestricted Kohn–Sham wave functions were expanded in a local atomic orbital basis. A numerical basis set was applied for the large system. The calculations were equipped with medium mesh and were all-electron ones. A self-consistent field procedure was carried out by applying a convergence criterion of 10^−5^ au to the energy and electron density. Integrating with the experimental results, the lattice constants and the conformation of adsorbed molecules were ulteriorly optimized. With the achievement of the density convergence criterion and energy requirements, we attained the optimized lattice constants and the intermolecular interaction energy between adsorbed molecules.

The model system presented the interactions between adsorbed molecules and the HOPG substrate. Owing to the similarity in the adsorption of adsorbed molecules containing a π-conjugated benzene ring on graphite and graphene, calculations were carried out on infinite graphene monolayers by utilizing PBC here. Along the normal direction, the interlayer spacing of graphene in the superlattice was 40 Å. As we built models for the adsorbates on graphene, graphene supercells were adopted, and the Brillouin zone was sampled using a 1 × 1 × 1 *k*-point mesh. The interaction energy (*E*_inter_) of the adsorbed molecules on graphite was given by *E*_inter_ = *E*_tot(adsorbates/graphene)_ − *E*_tot(isolated adsorbates in vacuum)_ − *E*_tot(graphene)_.

## 3. Results and discussion

### 3.1 Self-assembly nanostructure of H_4_BDETP

**Fig. 1:**
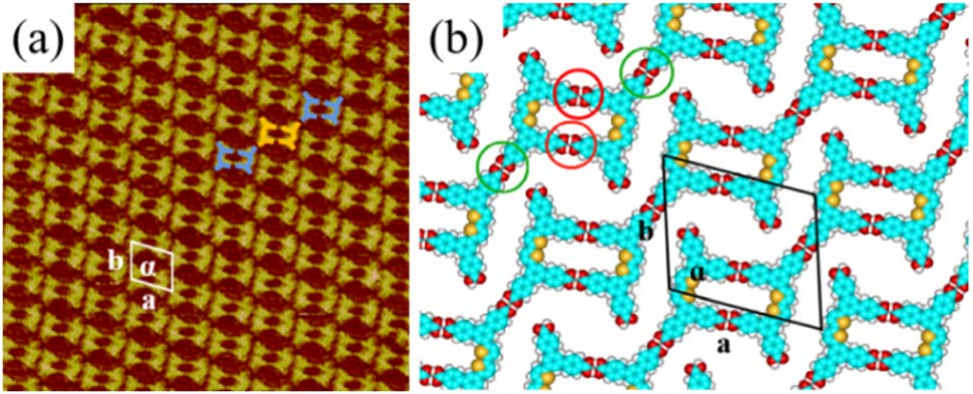
(a) High-resolution STM image of H_4_BDETP's linear structure at the 1-heptanoic acid/HOPG interface, and the tunneling conditions were: *I*_set_ = 216.7 pA, *V*_bias_ = 707.4 mV (32.0 nm × 32.0 nm). (b) The calculated molecular model for the linear structure.

In the self-assembly system of H_4_BDETP, a well-ordered linear nanostructure (Fig. S1†) was formed at the liquid/solid interface. Fig. 1(a) provides more details about the linear structure. It shows that arched bright spots represented H_4_BDETP molecules which adsorbed on the substrate in two different orientations. Two H_4_BDETP molecules marked by yellow lines were arranged in a face-to-face manner and constituted a dimer. In a dimer, the distance between the branch chain ends of the two molecules was about 0.2 ± 0.1 nm, which implied that there may be hydrogen bonds between their terminal carboxyl groups. The measured distance between the branch chain ends of the yellow dimer and the neighboring blue dimer was 0.3 ± 0.1 nm, and this indicated that hydrogen bonds may also have existed between them. The measured lattice parameters in Fig. 1(a) were: *a* = 3.5 ± 0.1 nm, *b* = 3.2 ± 0.1 nm, and *α* = 108 ± 1°. According to the optimized structural model (Fig. 1(b)), the two molecules in a dimer interacted with each other through two pairs of O–H⋯O hydrogen bonds labeled with red circles. At the same time, the H_4_BDETP dimer formed a pair of O–H⋯O hydrogen bonds with two neighboring dimers respectively, which were marked by green circles. And the two remaining carboxyl groups of the dimer were unsaturated. Table 1 shows that the calculated lattice parameters were in good agreement with the experimental data, indicating that the molecular packing model was reasonable.

**Table 1:** The experimental (exptl) and calculated (calcd) lattice parameters for the self-assembly structure of H_4_BDETP, two self-assembly structures of H_4_BTB, and the co-assembly structure of H_4_BDETP and H_4_BTB

		*a* (nm)	*b* (nm)	*α* (deg)
H_4_BDETP	Exptl	3.5 ± 0.1	3.2 ± 0.1	108 ± 1
	Calcd	3.42	3.1	108.4
H_4_BTB lamellar structure	Exptl	1.7 ± 0.1	3.6 ± 0.1	118 ± 1
	Calcd	1.65	3.7	118.5
H_4_BTB tetragonal structure	Exptl	1.6 ± 0.1	1.6 ± 0.1	95 ± 1
	Calcd	1.63	1.63	94.5
H_4_BDETP/H_4_BTB co-assembly structure	Exptl	7.8 ± 0.1	5.3 ± 0.1	45 ± 1
	Calcd	7.77	5.36	44.5

### 3.2 Self-assembly nanostructures of H_4_BTB

**Fig. 2:**
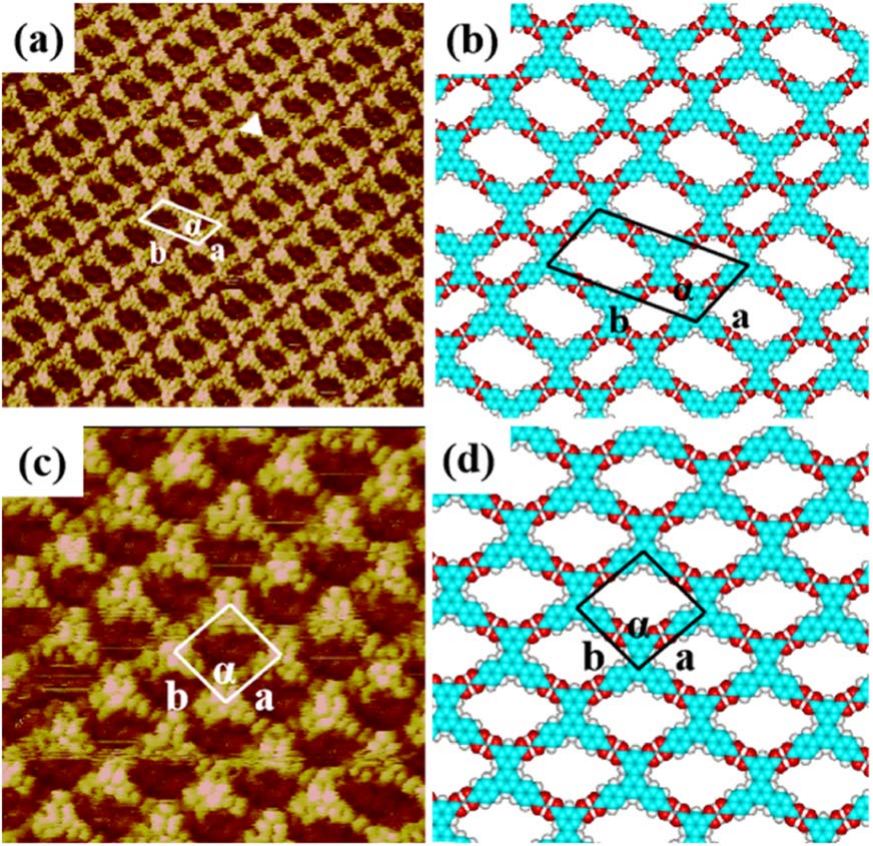
High-resolution STM image of H_4_BTB's (a) lamellar structure (25 nm × 25 nm) and (c) tetragonal structure (25 nm × 25 nm) at the 1-heptanoic acid/HOPG interface both with the tunneling conditions of *I*_set_ = 362.2 pA and *V*_bias_ = 668.0 mV. (b) and (d) The corresponding optimized molecular packing models for H_4_BTB's lamellar nanostructure in (a) and tetragonal nanostructure in (c) respectively.

When a droplet of H_4_BTB solution was dropped on the HOPG surface, H_4_BTB molecules aggregated into a lamellar structure (domain I) and tetragonal structure (domain II) as shown in Fig. S2(a) and (b).† In the high-resolution image of the lamellar structure (Fig. 2(a)), H_4_BTB molecules obviously appeared as triangle bright spots due to the high electron cloud density of the π-conjugated structure and were adsorbed on HOPG in two opposite orientations. One vertex of the H_4_BTB molecule labeled with a white triangle was adjacent to the vertices of the two H_4_BTB molecules, which implied that the *meta* carboxyl groups of the H_4_BTB molecule may form hydrogen bonds with the carboxyl groups of the adjacent molecules. Simultaneously, the two remaining vertices were also close to the vertices of another two molecules respectively, and this suggested that the two remaining terminal carboxyl groups also possibly formed hydrogen bonds with the carboxyl groups of other molecules. The unit cell was labeled with a white parallelogram in Fig. 2(a) and the lattice parameters were measured to be: *a* = 1.7 ± 0.1 nm, *b* = 3.6 ± 0.1 nm, and *α* = 118 ± 1°. Based on the STM results, the optimized model (Fig. 2(b)) of H_4_BTB's lamellar structure was provided by theoretical calculations. Fig. 2(b) shows that all carboxyl groups of H_4_BTB molecules interacted with the carboxyl groups of adjacent molecules *via* O–H⋯O hydrogen bonds. Every four H_4_BTB molecules with two orientations constituted a cavity, and two kinds of cavities were observed in the lamellar structure. As shown in Table 1, the calculated lattice parameters agreed well with the experimental parameters.

In the tetragonal structure of H_4_BTB (Fig. 2(c)), each triangle bright spot corresponded to one H_4_BTB molecule. Similar to the lamellar structure, the vertices of one H_4_BTB molecule in the tetragonal structure were adjacent to those of four H_4_BTB molecules, suggesting the existence of hydrogen bonds between their carboxyl groups. However, the difference from the lamellar structure was that all H_4_BTB molecules in the tetragonal structure were arranged in the same orientation and just formed one type of cavity. The measured parameters of the unit cell marked by white lines in Fig. 2(c) were: *a* = 1.6 ± 0.1 nm, *b* = 1.6 ± 0.1 nm, and *α* = 95 ± 1°. As shown in the optimized assembly model (Fig. 2(d)), all carboxyl groups of H_4_BTB molecules were saturated by forming O–H⋯O hydrogen bonds with the adjacent ones, which contributed to the generation of a tetragonal supramolecular network.

### 3.3 Co-assembly nanostructure of H_4_BDETP/H_4_BTB

To expand H_4_BDETP's packing structure, H_4_BTB as a bridging molecule was introduced into the pre-assembled structure of H_4_BDETP, and then characterized by STM. As displayed in (Fig. S3(a)–(c)†), a novel porous supramolecular network (domain I) different from the self-assembly nanostructures of H_4_BDETP or H_4_BTB was formed on the substrate. The formation of the H_4_BDETP/H_4_BTB co-assembly nanostructure was affected by the molar ratio of H_4_BDETP to H_4_BTB and the concentration of H_4_BTB to some extent. When the molar ratio of H_4_BDETP to H_4_BTB exceeded 2 : 1 (Fig. S3(a)†), H_4_BTB molecules disturbed a part of H_4_BDETP's self-assembly nanostructure and almost all H_4_BTB molecules formed a co-assembly nanostructure with H_4_BDETP. With the molar ratio reaching 2 : 1 (Fig. S3(b)†), the HOPG surface was nearly completely covered by the H_4_BDETP/H_4_BTB co-assembled nanostructure. As the concentration of H_4_BTB was increased and the molar ratio was controlled below 2 : 1 (Fig. S3(c)†), excessive H_4_BTB molecules formed a self-assembly structure that co-existed with the H_4_BDETP/H_4_BTB co-assembly nanostructure on HOPG.

**Fig. 3:**
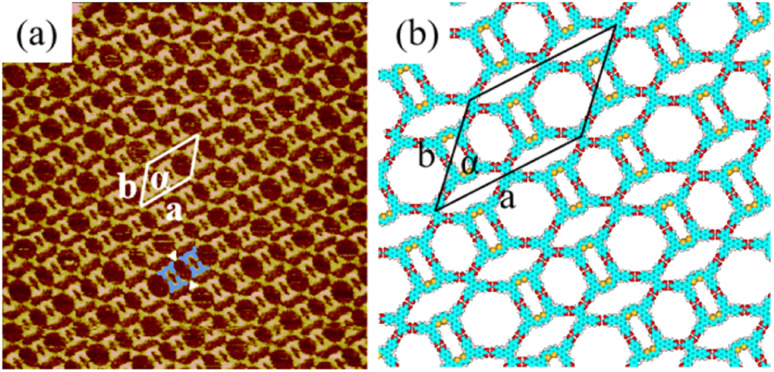
(a) High-resolution STM image of the H_4_BDETP/H_4_BTB co-assembly structure at the 1-heptanoic acid/HOPG interface, *I*_set_ = 265.5 pA and *V*_bias_ = 754.4 mV (46.3 nm × 46.3 nm). (b) The calculated molecular model for the H_4_BDETP/H_4_BTB co-assembly structure.

More details about the H_4_BDETP/H_4_BTB co-assembly nanostructure could be obtained from the high-resolution STM image (Fig. 3(a)). It showed that each arched bright spot represented one H_4_BDETP molecule, and every two H_4_BDETP molecules tended to constitute a dimer labeled with blue lines. Triangle bright spots representing H_4_BTB molecules isolated the H_4_BDETP dimers and increased the experimental unit cell parameters *a* = 7.8 ± 0.1 nm and *b* = 5.3 ± 0.1 nm respectively (Table 1). The optimized molecular model in Fig. 3(b) revealed that each H_4_BTB molecule was combined with four H_4_BDETP dimers *via* O–H⋯O hydrogen bonds between carboxyl groups. Every two H_4_BTB molecules with two different orientations and a pair of H_4_BDETP dimers constituted a hexagonal cavity. And the size of the hexagonal cavity depended on the position of H_4_BTB's carboxyl groups that formed hydrogen bonds with H_4_BDETP molecules.

To investigate the forming mechanism of the H_4_BDETP/H_4_BTB co-assembly nanostructure, DFT calculations were performed and energy values for the self-assembly nanostructures of H_4_BTB, H_4_BDETP and the co-assembly nanostructure of H_4_BDETP/H_4_BTB are presented in Table 2. The total energies included the energies of the interactions between molecules and the interactions between molecules and substrates. The thermodynamic stability can be evaluated by comparing the total energy of the distinct systems with the same lattice parameters. Due to the differences in the lattice parameters of systems, the total energy per unit area was adopted to compare the thermodynamic stability of distinct systems. The lower the total energy per unit area, the more stable the system was. As the total energies per unit area for the self-assembly nanostructures of H_4_BTB were lower than that for the self-assembly nanostructure of H_4_BDETP (Table 2), H_4_BTB could affect H_4_BDETP's linear structure, giving rise to the generation of the H_4_BDETP/H_4_BTB co-assembly nanostructure. The introduced H_4_BTB interacted with H_4_BDETP dimers through hydrogen bonds and saturated all carboxyl groups of H_4_BDETP dimers, which helped to reduce the energy of intermolecular interactions and led to a lower total energy per unit area of the H_4_BDETP/H_4_BTB co-assembly nanostructure (−0.276 kcal mol^−1^ Å^−2^) than that of H_4_BDETP's self-assembly nanostructure (−0.260 kcal mol^−1^ Å^−2^).

**Table 2:** Calculated total energy and total energy per unit area for the self-assembly structures of H_4_BDETP and H_4_BTB and the co-assembly structure of H_4_BDETP/H_4_BTB

	Interactions between molecules (kcal mol^−1^)	Interactions between molecules and substrate (kcal mol^−1^)	Total energy (kcal mol^−1^)	Total energy per unit area (kcal mol^−1^ Å^−2^)
H_4_BDETP	−79.737	−181.944	−261.681	−0.260
H_4_BTB lamellar structure	−118.258	−109.912	−228.170	−0.425
H_4_BTB tetragonal structure	−60.359	−52.624	−112.983	−0.426
H_4_BDETP/H_4_BTB co-assembly structure	−331.934	−473.123	−805.057	−0.276

It was interesting that the formation of the H_4_BDETP/H_4_BTB co-assembly nanostructure was associated with the deposition sequence. We adopted the opposite deposition sequence. When H_4_BDETP was introduced in the pre-assembled system of H_4_BTB, H_4_BTB's self-assembly nanostructure remained unchanged and the H_4_BDETP/H_4_BTB co-assembly nanostructure was not observed in Fig. S4(a)–(c),† despite the molar ratio of the two components being adjusted. The introduced H_4_BDETP was unable to break the pre-assembled nanostructure of H_4_BTB. Although the calculated energies of the intermolecular interactions and the molecules–substrate interactions for H_4_BTB's self-assembly nanostructure were higher than those for the H_4_BDETP/H_4_BTB co-assembly structure (Table 2), the smaller lattice parameters of H_4_BTB's self-assembly nanostructure (Table 1) lowered the total energies per unit area, which was thermodynamically unfavorable for the formation of the H_4_BDETP/H_4_BTB co-assembly nanostructure. The theoretical calculations were consistent with the experimental results showing that the sturdy pre-assembled nanostructure of H_4_BTB was unaffected by the introduced H_4_BDETP. We have also investigated the self-assembly behavior of the pre-mixed bi-component solution. Fig. S5(a) and (b)† show that no H_4_BDETP/H_4_BTB co-assembly nanostructure was formed except for the individual self-assembly nanostructures of H_4_BDETP and H_4_BTB. The above experimental observations indicated that the formation of the H_4_BDETP/H_4_BTB co-assembly nanostructure depended on the deposition sequence of the two components.

## 4. Conclusions

In conclusion, the self-assembly nanostructures of H_4_BDETP and H_4_BTB and their co-assembly nanostructure at the liquid/solid interface were investigated by STM. H_4_BDETP molecules aggregated into a linear nanostructure in the form of O–H⋯O hydrogen bonded dimers. And H_4_BTB molecules formed lamellar and tetragonal nanostructures. The introduced H_4_BTB molecules disturbed the intermolecular O–H⋯O hydrogen bonds between H_4_BDETP dimers and formed new O–H⋯O hydrogen bonds with H_4_BDETP dimers, contributing to the generation of a H_4_BDETP/H_4_BTB co-assembly nanostructure. Notably, the H_4_BDETP/H_4_BTB co-assembly nanostructure was not observed when the deposition sequence of H_4_BDETP and H_4_BTB on HOPG was altered. This work was interesting and may have an enlightening significance for selecting bridging molecules to expand the supramolecular nanostructure of carboxylic acid derivatives and help deepen the understanding of the formation of binary supramolecular nanostructures.

## Author contributions

Xuan Peng and Linlin Gan contributed to this work equally.

## Conflicts of interest

There are no conflicts to declare.

## Supplementary Material

NA-005-D3NA00389B-s001

## References

[cit1] Nickmans K., Schenning A. P. (2018). Adv. Mater..

[cit2] Saraswathi S. K., Joseph J. (2022). ACS Appl. Nano Mater..

[cit3] Shin D. O., Mun J. H., Hwang G.-T., Yoon J. M., Kim J. Y., Yun J. M., Yang Y.-B., Oh Y., Lee J. Y., Shin J. (2013). ACS Nano.

[cit4] King N. P., Bale J. B., Sheffler W., McNamara D. E., Gonen S., Gonen T., Yeates T. O., Baker D. (2014). Nature.

[cit5] Okesola B. O., Mata A. (2018). Chem. Soc. Rev..

[cit6] Li L., Sun R., Zheng R. (2021). Mater. Des..

[cit7] Mali K. S., Teyssandier J., Bilbao N., De Feyter S. (2022). Supramol. Chem..

[cit8] Velpula G., Takeda T., Adisoejoso J., Inukai K., Tahara K., Mali K. S., Tobe Y., De Feyter S. (2017). Chem. Commun..

[cit9] Fang J., Zhu X., Luo W., Shi J., Wang L., Tu B., Zeng Q., Xiao X. (2022). Chin. Chem. Lett..

[cit10] Xiao Y., Cheng L., Sui X., Wang Q., Chen J., Deng D., Zhang J., Peng X., Li X., Xiao X. (2022). Nano Res..

[cit11] Xiao Y., Cai F., Peng X., Kang X., Lei P., Li X., Xu H., Xiao X., Tu B., Zeng Q. (2021). Chin. Chem. Lett..

[cit12] Peng X., Meng T., Wang L., Cheng L., Zhai W., Deng K., Ma C.-Q., Zeng Q. (2023). Chin. Chem. Lett..

[cit13] Feng Z., Cudia C. C., Floreano L., Morgante A., Comelli G., Dri C., Cossaro A. (2015). Chem. Commun..

[cit14] Liu Y., Wang Y., Zhang S., Zou H., Miao X., Deng W. (2023). J. Phys. Chem. C.

[cit15] Peng X., Xiao Y., Mu B., Deng K., Tian W., Xiao X., Li X., Zeng Q. (2021). Appl. Surf. Sci..

[cit16] Zhang X., Li N., Gu G.-C., Wang H., Nieckarz D., Szabelski P., He Y., Wang Y., Xie C., Shen Z.-Y. (2015). ACS Nano.

[cit17] Zhang X., Ding H., Yang S., Yang H., Yang X., Li B., Xing X., Sun Y., Gu G., Chen X. (2023). Small.

[cit18] Xie Y., Liu C., Cheng L., Fan Y., Li H., Liu W., Zhu L., Li X., Deng K., Zeng Q. (2022). Chin. Chem. Lett..

[cit19] Li X., Li J., Ma C., Chen C., Zhang S., Tu B., Duan W., Zeng Q. (2021). Chin. Chem. Lett..

[cit20] Li J., Luo W., Zhang S., Ma C., Xiao X., Duan W., Zeng Q. (2022). Nano Res..

[cit21] Cai L., Wang L., Kang S., Geng Y., Deng K., Zheng Q., Zeng Q. (2016). J. Phys. Chem. C.

[cit22] Cometto F., Frank K., Stel B., Arisnabarreta N., Kern K., Lingenfelder M. (2017). Chem. Commun..

[cit23] Meng T., Lei P., Zhang Y., Deng K., Xiao X., Zeng Q. (2022). Chin. J. Chem..

[cit24] Pinfold H., Greenland C., Pattison G., Costantini G. (2020). Chem. Commun..

[cit25] Lei P., Ma L., Zhang S., Li J., Gan L., Deng K., Duan W., Li W., Zeng Q. (2022). Chin. Chem. Lett..

[cit26] Peng X., Cheng L., Zhu X., Geng Y., Zhao F., Hu K., Guo X., Deng K., Zeng Q. (2018). Nano Res..

[cit27] Kampschulte L., Werblowsky T. L., Kishore R. S., Schmittel M., Heckl W. M., Lackinger M. (2008). J. Am. Chem. Soc..

[cit28] Yan L., Kuang G., Lin N. (2018). Chem. Commun..

[cit29] Zhang S., Li J., Gan L., Ma L., Ma W., Zhang M., Cheng F., Deng K., Zeng Q. (2023). Nanoscale.

[cit30] Li W., Xu S., Chen X., Xu C. (2021). Chin. Chem. Lett..

[cit31] Liang Q., Yu Y., Feng G., Shen Y., Yang L., Lei S. (2020). Chem. Commun..

[cit32] Delley B. (2000). J. Chem. Phys..

[cit33] Perdew J. P., Wang Y. (1992). Phys. Rev. B: Condens. Matter Mater. Phys..

[cit34] Perdew J. P., Burke K., Ernzerhof M. (1996). Phys. Rev. Lett..

