# Supplementary material for: Two-dimensional self-assembly and co-assembly of two tetracarboxylic acid derivatives investigated by STM
Source: Nanoscale Adv. 2023 Aug 3;5(18):4752–7. doi: 10.1039/d3na00389b (PMC10496876; doi:10.1039/d3na00389b)
Supplement: NA-005-D3NA00389B-s001 [file NA-005-D3NA00389B-s001.pdf]

Electronic Supplementary Material (ESI) for Nanoscale Advances.
This journal is © The Royal Society of Chemistry 2023

Electronic Supplementary Information for

Two-dimensional self-assembly and co-assembly of two tetracarboxylic acid derivatives investigated by STM

Xuan Peng,[a, d, ξ] Linlin Gan,[a, c, ξ] Wenchao Zhai,[d] Xiaoling Chen,[d] Ke Deng,[*a] Wubiao Duan,[*c] Wei Li,[*d] Qingdao Zeng[*a, b]

a. *CAS key Laboratory of Standardization and Measurement for Nanotechnology, CAS Center for Excellence in Nanoscience, National Center for Nanoscience and Technology (NCNST), Beijing 100190, China.*
b. *Center of Materials Science and Optoelectronics Engineering, University of Chinese Academy of Sciences, Beijing 100049, China.*
c. *Department of Chemistry, School of Science, Beijing Jiaotong University, Beijing 100044, China.*
d. *School of Science, Nanchang Institute of Technology, Nanchang 330099, China.*

*Corresponding author E-mail: kdeng@nanoctr.cn; liweidting@nit.edu.cn; wbduan@bjtu.edu.cn; zengqd@nanoctr.cn.

[ξ] Xuan Peng and Linlin Gan contributed to this work equally.

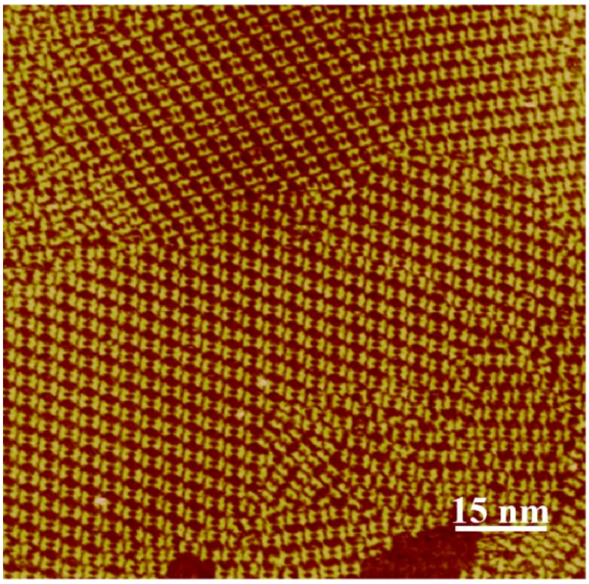

Fig. S1 Large-scale STM image of H_4BDETP's self-assembled nanostructure at the 1-heptanoic acid/HOPG interface with the tunneling conditions of I_{set} = 216.7 pA, V_{bias} = 707.4 mV.

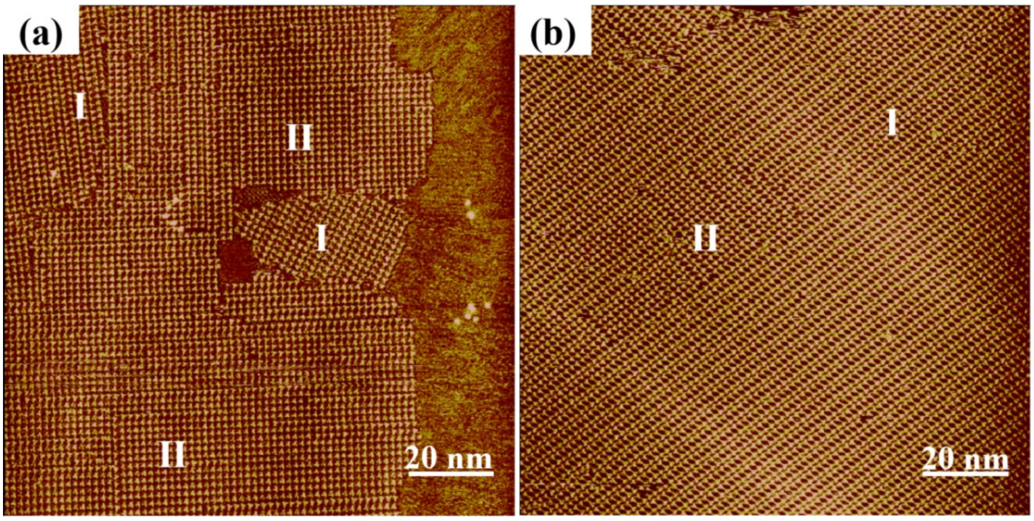

Fig. S2 (a), (b) Large-scale STM images of H_4BTB's two self-assembled nanostructure (lamellar nanostructure in domain I and tetragonal nanostructure in domain II) at the 1-heptanoic acid/HOPG interface, both the tunneling conditions were: I_{set} = 363.2 pA, V_{bias} = 668.0 mV.

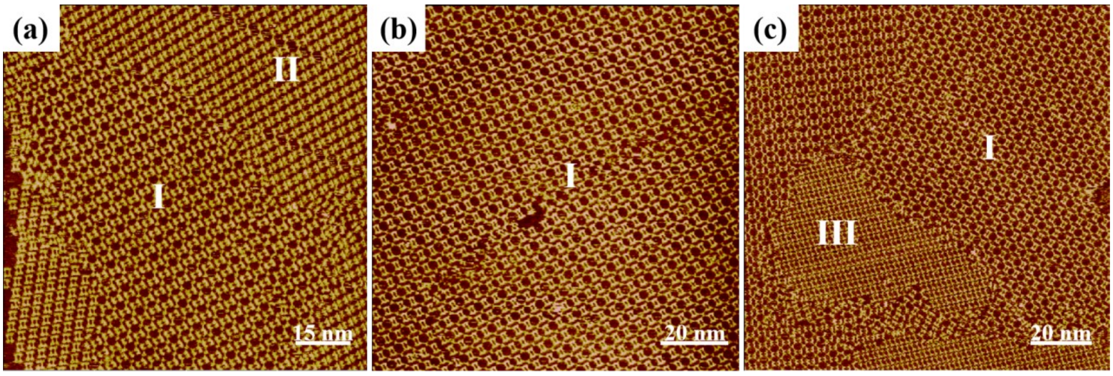

Fig. S3 Large-scale STM images of two-component system prepared by dropping H_4BTB to pre-assembled H_4BDETP with different molar ratios: (a) H_4BDETP: H_4BTB > 2:1, I_{set} = 219.7 pA , V_{bias} = 655.2 mV; (b) H_4BDETP: H_4BTB = 2:1, I_{set} = 265.5 pA, V_{bias} = 754.4 mV; (c) H_4BDETP: H_4BTB < 2:1, I_{set} = 174.0 pA, V_{bias} = 551.5 mV. (Domain I: H_4BDETP/H_4BTB co-assembly structure. Domain II: self-assembly structure of H_4BDETP. Domain III: self-assembly structure of H_4BTB)

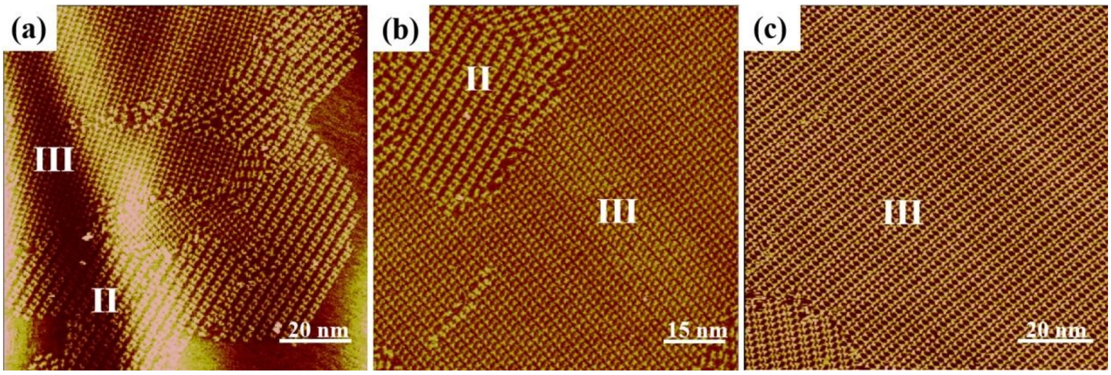

Fig. S4 Large-scale STM images of assembled structures prepared by dropping H_4BDETP to pre-assembled H_4BTB with different molar ratios: (a) H_4BDETP: H_4BTB > 2:1, I_{set} =296.0 pA, V_{bias} = 578.9 mV; (b) H_4BDETP: H_4BTB = 2:1, I_{set} = 296.0 pA, V_{bias} = 578.9 mV; (c) H_4BDETP: H_4BTB < 2:1, I_{set} = 299.8 pA, V_{bias} = 699.8 mV. (Domain II: self-assembly structure of H_4BDETP. Domain III: self-assembly structure of H_4BTB)

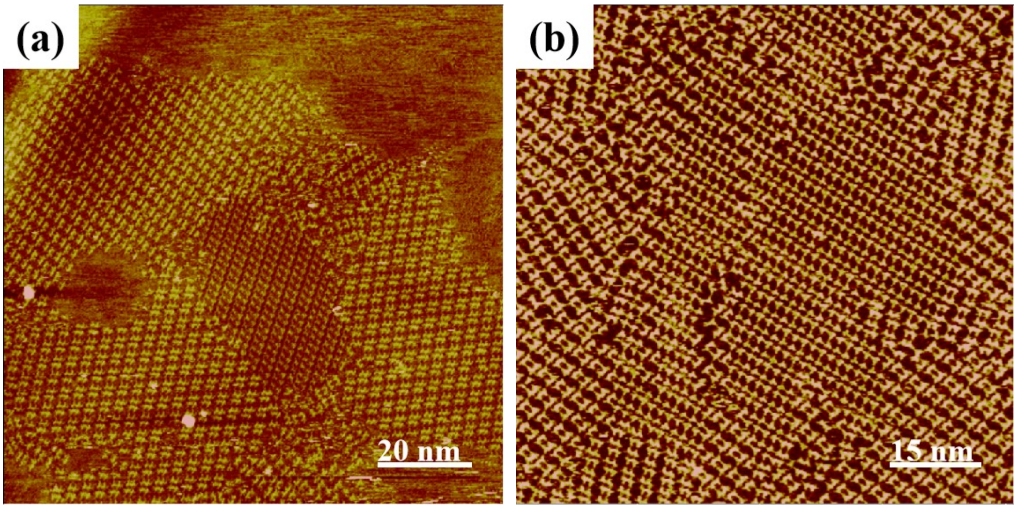

Fig. S5 Large-scale STM images of two-component system prepared by pre-mixing H_4BTB and H_4BDETP solution：(a) I_{set} = 241.1 pA, V_{bias} = 846.3 mV , (b) I_{set} = 335.7 pA, V_{bias} = 660.4 mV . The concentration of H_4BTB increased from (a) to (b).
